# Neurobehavioral Precursors of Compulsive Cocaine Seeking in Dual Frontostriatal Circuits

**DOI:** 10.1016/j.bpsgos.2023.06.001

**Published:** 2023-06-22

**Authors:** Jolyon A. Jones, Aude Belin-Rauscent, Bianca Jupp, Maxime Fouyssac, Stephen J. Sawiak, Katharina Zuhlsdorff, Peter Zhukovsky, Lara Hebdon, Clara Velazquez Sanchez, Trevor W. Robbins, Barry J. Everitt, David Belin, Jeffrey W. Dalley

**Affiliations:** a Department of Psychology, University of Cambridge, Cambridge, United Kingdom; b Behavioural and Clinical Neuroscience Institute, University of Cambridge, Downing Site, Cambridge, United Kingdom; c Department of Neurosciences, Central Clinical School, Monash University, Melbourne, Victoria, Australia; d Department of Physiology, Development and Neuroscience, University of Cambridge, Cambridge, United Kingdom; e Centre for Addiction and Mental Health, Toronto, Ontario, Canada; f Department of Psychiatry, Herschel Smith Building for Brain and Mind Sciences, Forvie Site, Cambridge, United Kingdom

**Keywords:** Addiction, Cocaine, Compulsivity, Endophenotype, Impulsivity, MRI, Stickiness

## Abstract

**Background:**

Only some individuals who use drugs recreationally eventually develop a substance use disorder, characterized in part by the rigid engagement in drug foraging behavior (drug seeking), which is often maintained in the face of adverse consequences (i.e., is compulsive). The neurobehavioral determinants of this individual vulnerability have not been fully elucidated.

**Methods:**

Using a prospective longitudinal study involving 39 male rats, we combined multidimensional characterization of behavioral traits of vulnerability to stimulant use disorder (impulsivity and stickiness) and resilience (sign tracking and sensation seeking/locomotor reactivity to novelty) with magnetic resonance imaging to identify the structural and functional brain correlates of the later emergence of compulsive drug seeking in drug-naïve subjects. We developed a novel behavioral procedure to investigate the individual tendency to persist in drug-seeking behavior in the face of punishment in a drug-free state in subjects with a prolonged history of cocaine seeking under the control of the conditioned reinforcing properties of a drug-paired Pavlovian conditioned stimulus.

**Results:**

In drug-naïve rats, the tendency to develop compulsive cocaine seeking was characterized by behavioral stickiness–related functional hypoconnectivity between the prefrontal cortex and posterior dorsomedial striatum in combination with impulsivity-related structural alterations in the infralimbic cortex, anterior insula, and nucleus accumbens.

**Conclusions:**

These findings show that the vulnerability to developing compulsive cocaine-seeking behavior stems from preexisting structural or functional changes in two distinct corticostriatal systems that underlie deficits in impulse control and goal-directed behavior.

Compulsivity is a core component of addictive behavior, which is defined by drug seeking and taking that persists despite personal harm (1, 2, 3). Vulnerability to compulsivity is hypothesized to result from an interaction between preexisting individual differences in behavioral traits, environmental and experiential factors, and long-term drug exposure (4, 5, 6). Stimulant use disorder in humans has been associated with novelty seeking (7), impulsivity (8, 9, 10), and impaired cognitive control (11) alongside alterations in both ventral and dorsal corticostriatal circuits (10,12,13). However, whether these neurobehavioral correlates emerge as a consequence of long-term exposure to the drug (8,14, 15, 16, 17) or are instead preexisting risk factors for vulnerability that contribute to the emergence of compulsive drug use has yet to be determined.

Studies conducted with rodents have implicated causal neural substrates of impulsivity and novelty preference in vulnerability to compulsivity (4,18,19). By contrast, novelty reactivity and sign tracking (the tendency to attribute incentive salience to Pavlovian cues) (20) confer increased susceptibility to the acquisition of cocaine self-administration and sensitivity to the associative properties of cocaine-associated cues, respectively, rather than vulnerability to addiction (4,19,21).

However, none of these studies has acknowledged that compulsive drug use conflates phases of drug foraging or anticipation, which actually occupy most of the time spent in waking activity by addicted individuals, prior to actual drug taking (i.e., consummatory behavior, operationalized as intravenous self-administration under a fixed-ratio schedule of reinforcement in laboratory animals). In addition, in everyday life, Pavlovian conditioned stimulus (CS)–dependent motivational and instrumental mechanisms interact to maintain drug-seeking behavior over prolonged periods whereby response-produced, drug-paired CSs bridge attendant delays to eventual drug use through conditioned reinforcement (22,23).

Thus, studies of compulsivity in stimulant use disorder that have been conducted to date have investigated neurobehavioral circuitries implicated in compulsive stimulant drug taking (24, 25, 26) following drug exposure, thereby limiting our understanding of the neuropsychological basis of vulnerability to the tendency to compulsively seek drugs over prolonged periods of time.

In preclinical models, compulsive drug use has generally been assessed in terms of the resistance to punishment of drug self-administration (27). Such self-administration behaviors exemplify instrumental responding governed by the principles of reinforcement learning. However, the propensity for compulsive drug seeking in terms of possible predisposing differences in reinforcement sensitivity and the balance between goal-directed and habitual control over behavior (28,29) have not yet been compared in conjunction with Pavlovian or neurobehavioral vulnerability markers.

Consequently, we combined a multidimensional behavioral approach to vulnerability with magnetic resonance imaging (MRI) to identify the structural and functional brain correlates of the later emergence of compulsive cue-controlled drug seeking in drug-naïve male rats. We developed a novel behavioral procedure to investigate the individual vulnerability to persist in seeking cocaine in the face of punishment in individuals with a prolonged history of cue-controlled cocaine seeking (30) (Figure S1).

## Methods and Materials

### Subjects and Timeline of the Experiment

As described in more detail in the Supplement, male rats underwent several MRI scans before being food restricted to 85% of their free-feeding weight and screened for behavioral endophenotypes of vulnerability or resilience to cocaine addiction (4,31). As summarized in Figure S1, rats were first screened for the trait of sign tracking (20) and then impulsivity (19), reversal learning (16), and locomotor reactivity to novelty (32). Individual differences in approach responses to CSs (e.g., sign- or goal-tracking trait) were assessed using an autoshaping task (4,20). Impulsivity was measured with the 5-choice serial reaction time task (33). A spatial reversal learning task was used to measure reinforcement learning (as assessed by the ability to learn to update contingencies under probabilistic reversal conditions) and stickiness (34). Stickiness reflects the tendency to adopt and follow an internal rule driven by an overall outcome but not the immediate consequences of each response under probabilistic reversal conditions, as manifested by the tendency to stick to the response from one trial to the next regardless of the immediate outcome. Locomotor reactivity to novelty was assessed using 4 open fields and a video tracking system (ViewPoint Behavior Technology) (4). On completion of behavioral phenotyping, on postnatal day 212 to 244, rats underwent MRI scans prior to intravenous catheter surgery, after which they were singly housed for the duration of the experiment. Rats were then trained to seek cocaine in the presence of the response-contingent drug-paired CS under a fixed interval 15 minutes (fixed ratio 10:S) second-order schedule of reinforcement, as previously described (35) and detailed in the Supplement. After 20 daily sessions of cue-controlled cocaine seeking, conditions previously shown to result in the development of incentive habits (35), the tendency to persist in seeking cocaine despite adverse consequences was assessed over 5 sessions by the resistance of drug seeking to contingent mild electric foot shocks, as described in detail in the Supplement. All experiments were carried out in accordance with the (U.K. Animals) Scientific Procedures Act (1986) under U.K. Home Office project licenses PPL 70/7587 and PPL 70/8072 held by BJ and DB, respectively, and were approved by the University of Cambridge Ethics Committee. The number of animals used during each stage of this longitudinal study is summarized in Tables S1 and S2.

### Drugs

Cocaine hydrochloride (kindly supplied by the National Institute on Drug Abuse Drug Supply Program to DB) was dissolved in sterile 0.9% saline. Drug doses are reported as the salt form.

### Magnetic Resonance Imaging

#### Imaging Acquisition

High-resolution MRI was performed on a 9.4T horizontal bore MRI system (Bruker BioSpec 94/20 Bruker Ltd.). Images were acquired under isoflurane anesthesia, as previously described (25), using the manufacturer-supplied rat brain array coil with the rat in a prone position, as described in detail in the Supplement. The structural and functional imaging data used in this study were obtained with an MRI scan administered between behavioral screening and cocaine self-administration.

#### Voxel-Based Morphometry

Voxel-based morphometry was performed to assess morphological correlates of impulsivity, stickiness, and compulsivity. An unbiased whole-brain analysis approach was adopted to capture brain-wide significant differences in gray matter, as described in the Supplement. Images were first manually reoriented to match the orientation of a reference template image using the bulk manual registration tool in SPM8 (Wellcome Trust Centre for Neuroimaging, University College London) toolbox SPMMouse (SPMMouse, Wolfson Brain Imaging Centre, University of Cambridge) (36). After correspondence had been achieved, structural images were bias corrected and segmented into 3 different tissue classes (gray, white, and cerebrospinal fluid). Tissue class images were then rigidly coregistered to a reference template image. Nonlinear registration was achieved through the use of the DARTEL procedure (37). Using the DARTEL warp fields, images were then warped and modulated to match the newly generated template images (Figure S2). All images were manually checked for accurate registration and segmentation. Modulated gray matter maps were smoothed with an isotropic Gaussian kernel of 0.45 mm to promote normality of the data and mitigate imperfections in image registration. As detailed in the Supplement, a general linear model was used for voxelwise analysis on the smoothed maps. Three independent models were created with block designs to assess main effects of impulsivity (high-impulsivity [HI] rats, *n* = 12; low-impulsivity [LI] rats, *n* = 12), compulsivity (high-compulsivity [HC] rats, *n* = 7; low-compulsivity [LC] rats, *n* = 7), and stickiness (rats with high kappa, *n* = 14 or low kappa, *n* = 14). Two independent linear regression models were created to identify the neural corelates of compulsive cocaine seeking (*n* = 39) and cocaine seeking under no punishment (*n* = 39).

Main effects of these linear regression models based on the entire cohort of 39 individuals were further assessed with contrasts based on Student’s *t* tests, with a restricted number of assessments made to avoid type I errors due to multiple comparisons: 1) HI < LI, 2) HC < LC, 3) HC > LC, 4) high kappa < low kappa, 5) compulsive cocaine seeking (negative correlation), and 6) cocaine seeking under no punishment (negative correlation), among others (Figure S3). These were selected based on our previous findings that HI was related to reduced gray matter volume in the ventral striatum (38) and thinning of the insular cortex (39), and evidence of gray matter abnormalities in stimulant-dependent individuals (40,41). To control for multiple comparisons across voxels, cluster statistics were used. A cluster-forming threshold of *p* < .005 was used to generate clusters, which were then considered further when *p* < .05 (uncorrected cluster-level significance), as previously described (42,43). In addition, small clusters (smaller than a 0.5-mm sphere equivalent, ∼130 voxels) were ignored to further mitigate type I errors.

#### Functional Connectivity Analysis

Prior to functional connectivity analysis, voxel dimensions in the header files for both the structural (magnetization transfer–weighted) and functional images were scaled by a factor of 10 to facilitate processing with software designed for human brain images. After preprocessing (see the Supplement for more details) (44), images were first manually reoriented to match a reference template as described above and processed as described in the Supplement. Registration accuracy was manually checked for each image (Figure S4). Temporal spikes were then removed (3dDespike), followed by motion correction (3dvolreg). Excess motion was calculated through relative framewise displacement as described in the Supplement. To assess any residual motion effects, average framewise displacement was regressed against global connectivity (calculated as the average correlation value for each subject’s correlation matrix), regional connectivity (average row-wise correlation value for each subject’s correlation matrix), and edgewise connectivity (correlation between each region-to-region value for each subject). No connectivity measure was related to average framewise displacement (Figure S5). Signal-to-noise ratio (SNR) and temporal SNR were also calculated for each image, with SNR and temporal SNR being very consistent with those in previously published reports (44) (Figure S5). Following preprocessing, region-to-region functional analysis was carried out. The first eigenvariate of the blood oxygen level–dependent time series was extracted for each region of interest (Figure S6) (fslmeants), and the Spearman’s rho correlation coefficient was calculated pairwise between each region of interest as a measure of functional connectivity (Figure S3). Correlation matrices were subsequently used to investigate the relationship between functional connectivity and behavior. All correlations between region of interest blood oxygen level–dependent signals were corrected for multiple comparisons with a false discovery rate set at *q* = .05 (45).

### Data and Statistical Analyses

Data are presented as means ± 1 SEM, box plots (medians ± 25% and minimum/maximum as whiskers), or individual data points and were analyzed using STATISCA 10 (StatSoft) as described in detail in the Supplement. When the assumptions of a normal distribution or homogeneity of variance were significantly violated, the data were log-transformed. Behavioral data were subjected to repeated-measures analysis of variance. Significant interactions were analyzed further using the Newman-Keuls post hoc test or hypothesis-driven planned comparisons whenever appropriate. For all analyses, significance was set at α = 0.05. Effect sizes are reported as partial eta squared values (η_p_^2^).

Dimensional relationships between behavioral variables were analyzed using Pearson’s correlation coefficient, *r*. Following dimension reduction, as detailed in the Supplement, descriptive statistical analyses (factorial analyses) were carried out on variables with inherent theoretical construct such as premature responses during long-intertrial-interval sessions in the 5-choice serial reaction time task (33) (Tables S1 and S2) using a principal component extraction method with the maximum number of factors set at n−1, where n refers to the number of variables used in the analysis, with a minimum eigenvalue of 1 and varimax rotation.

## Results

Following acquisition of intravenous cocaine self-administration, rats were progressively trained to seek cocaine daily for long (15 minutes) periods of time under the control of the conditioned reinforcing properties of the response-produced, drug-paired CS under a fixed interval 15 minutes (fixed ratio 10:S) second-order schedule of reinforcement (22). The compulsive nature of cocaine seeking was then measured individually as the tendency to persist in seeking cocaine, in a drug-free state, in the face of response-contingent mild foot-shock punishment.

**Figure 1:**
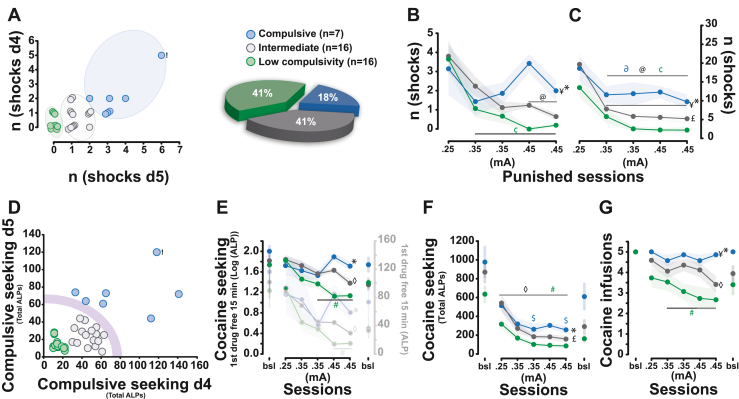
Emergence of a compulsive foraging phenotype in a subpopulation of rats with a long history of cue-controlled cocaine seeking. **(A)** Rats with a long history of cocaine seeking (*n* = 39) were identified by clustering as HC, Int, and LC rats based on the number of shocks they tolerated to pursue cocaine seeking over successive daily 15-minute drug-free periods. Under the threat of punishment, HC rats received more foot shocks than Int and LC rats during the drug-free seeking periods **(B)** and the total session **(C)** (effect of compulsivity: *F*_2,36_ = 3.31, *p* = .047, η_p_^2^ = 0.15 and *F*_2,36_ = 7.59, *p* = .0018, η_p_^2^ = 0.29, respectively; compulsivity × session interaction: *F*_8,144_ = 2.15, *p* = .035, η_p_^2^ = 0.11 and *F*_8,144_ < 1, respectively). **(D)** This willingness to persist in seeking cocaine in the face of punishment represented a break in the continuum between LC and Int rats on the one hand and the heterogeneous compulsive population on the other (represented as a purple arc), reflected in higher levels of responding on the active lever in HC than LC rats during the first drug-free intervals (*F*_2,36_ = 3.29, *p* = .048, η_p_^2^ = 0.15) **(E)** and the entire sessions **(F)** (*F*_2,36_ = 4.18, *p* = .023, η_p_^2^ = 0.19). **(G)** HC rats did not decrease their cocaine intake in the face of punishment as LC and Int rats did (effect of compulsivity: *F*_2,36_ = 4.10, *p* = .025, η_p_^2^ = 0.18). n (shocks d4) and n (shocks d5) refer to the number of shocks on the fourth and fifth punishment session, respectively. n (shocks) refers to the total number of shocks. Newman-Keuls post hoc tests, #, $, ◊: LC, HC, Int different from baseline, *p* < .05, respectively; ¢, ∂, @: LC, HC, Int different from d1, *p* < .05, respectively; ∗,¥: HC different from LC and Int, *p* < .05, respectively; £: Int different from LC, *p* < .05. ALP, active lever press; bsl, baseline; HC, high-compulsivity; Int, intermediate-compulsivity; LC, low-compulsivity.

HC, LC, and intermediate-compulsivity (IC) rats identified according to an unbiased cluster analysis of the punishment resistance of cocaine seeking (46) represented 18%, 41%, and 41% of the cohort, respectively (Figure 1A). HC rats originated from 5 litters, while IC and LC rats originated from 9 and 11 separate litters, respectively. Of these, 4 HC, 11 IC, and 7 LC rats were siblings from 2, 4, and 3 independent litters, respectively. The incidence of HC rats, of which the genetic contribution accounted for only a 14.5% increased risk (Bayes’ law), is similar to that previously reported for compulsive cocaine taking (4) and the prevalence of addiction in human cocaine users (47). HC rats received more shocks than the other groups during both the first 15-minute drug-free period (Figure 1B) and the entire daily sessions (Figure 1C). This reflected a break in the continuum of the tendency to persist in cocaine seeking under the threat of punishment (Figure 1D), similar to compulsive alcohol seeking (48) and cocaine self-administration (18). This was demonstrated by HC rats maintaining higher levels of responding on the active lever than the other groups during the first drug-free seeking period (Figure 1E) and throughout each punished session (Figure 1F). Consequently, although only seeking (and not taking) behavior was punished, noncompulsive rats decreased their cocaine intake significantly across punished sessions, whereas HC rats continued to obtain the maximum number of cocaine infusions available daily (Figure 1G). The development of punishment-resistant, cue-controlled cocaine seeking shown by HC rats, which was replicated in an independent cohort (Figure S7), was neither due to differences in their acquisition of intravenous drug self-administration or drug seeking compared with LC rats (Figure S8) nor due to a differential sensitivity to pain as assessed using a hot plate test (Figure S7) (4).

**Figure 2:**
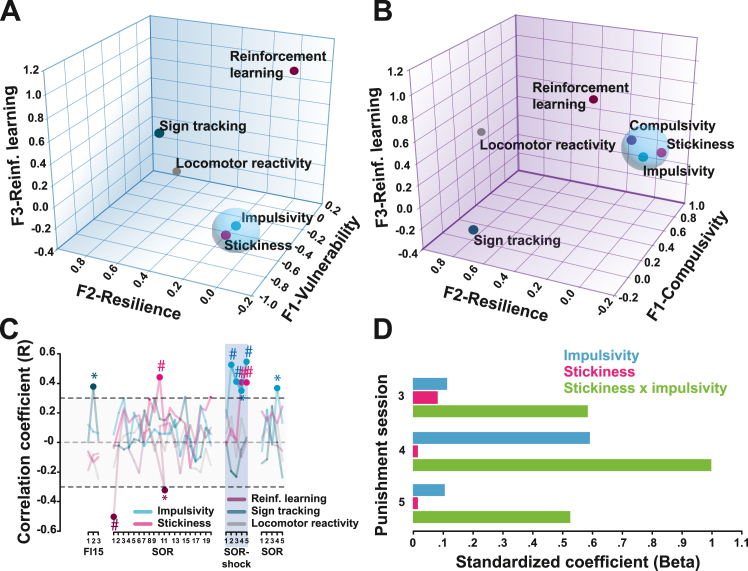
Impulsivity and stickiness interact to confer increased vulnerability to develop compulsive cocaine seeking. **(A)** A principal component analysis using locomotor reactivity to novelty, sign tracking, the factor of reinforcement learning αβ, the stickiness factor κ, and impulsivity revealed 3 overarching factors accounting for >72.9% of the total variance (see Table S1 for more details). Impulsivity and stickiness loaded onto factor 1, which represents vulnerability to compulsivity and was orthogonal to factor 2, which by accounting for sign tracking and locomotor reactivity to novelty, represents resilience to compulsivity. Finally, reinforcement learning (αβ) loaded on the third, independent factor. **(B)** When compulsive cocaine seeking was added to the model, it loaded on the same factor as impulsivity and stickiness, while novelty reactivity and sign tracking remained clustered on an orthogonal factorial capturing resilience, and reinforcement learning (αβ) was accounted for by a third independent factor. **(C)** Further dimensional analyses revealed that impulsivity and stickiness were only correlated with cocaine seeking when under punishment, even though κ tended to correlate with cue-controlled cocaine seeking at baseline prior to the introduction of punishment. This suggests a relationship between stickiness and the tendency to engage in cue-controlled cocaine seeking. Gray area reflects the −0.25 to +0.25 marginal *R* value range. **(D)** Factorial regression analysis demonstrated that stickiness (κ) interacted with impulsivity in better predicting compulsive cocaine seeking on each of the last 3 days of punishment. ^#^*p* < .01, *∗p* < .05. F, factor; FI15, fixed interval 15 minutes; Reinf., reinforcement; SOR, second-order schedule of reinforcement.

Factorial analysis building on a dimension reduction strategy (see the Supplement) was used retrospectively on the putative behavioral markers of vulnerability (Figure S9) to identify the detailed phenotype of drug-naïve rats that later developed compulsive cue-controlled drug seeking (Figure 2). These markers were premature responses in the 5-choice serial reaction time task (49), indexing impulsivity; the locomotor response to novelty (19), indexing novelty reactivity; conditioned approach to a food-related CS, indexing sign tracking (4); and reinforcement learning parameters (50) derived from a serial reversal learning task reflecting reward-based learning (α), reinforcement sensitivity (β), and response stickiness (κ) (16). The latter parameter is the likelihood of the same response being repeated regardless of its reinforcing outcome and is thus a putative measure of value-free habitual responding (51). Importantly, this stickiness is elevated in humans with stimulant use disorder (52), but it is unknown whether it predisposes to addiction.

Impulsivity and stickiness loaded on factor 1 of the analysis (Figure 2A; Table S3) while sign tracking and novelty reactivity loaded on factor 2, independent of factor 3, which accounted for α and β (see the Supplement for more details). This multidimensional behavioral structure showed that more than half of the overall model variance was explained by factor 1 (vulnerability to compulsivity) and factor 2 (resilience to compulsivity). Additional factorial analysis incorporating compulsive cocaine seeking into this model (Figure 2B; Table S4) revealed a shared construct of impulsivity, stickiness, and compulsive cocaine seeking represented by factor 1, whereas factors 2 and 3 accounted for resilience and reinforcement learning parameters as in the initial analysis.

Subsequently, we found that impulsivity was correlated with cue-controlled cocaine seeking only when rats had learned that persisting in responding resulted in punishment, i.e., from the second punished session onward (Figure 2C). By contrast, stickiness was not only correlated with cocaine seeking under punishment, although less systematically and robustly, but it was also marginally related to baseline levels of cue-controlled cocaine seeking (Figure 2C). Further analysis revealed that the co-occurrence of the behavioral traits of impulsivity and stickiness resulted in an increased vulnerability to developing compulsive cocaine seeking, the product of their variance systematically being a better predictor of compulsivity than that of each alone (Figure 2D).

**Figure 3:**
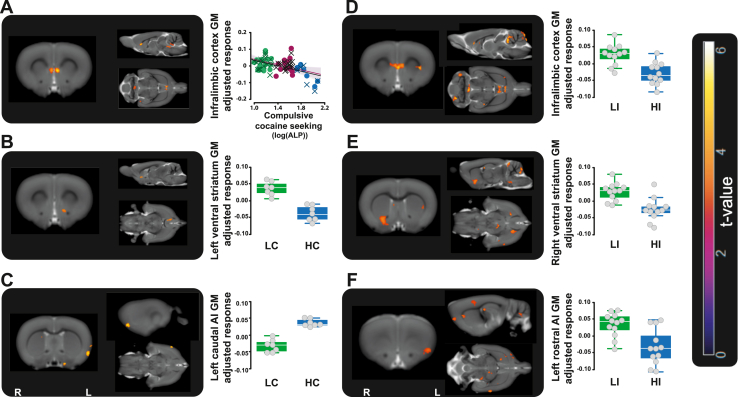
GM alterations in the prefrontal cortex, insula, and ventral striatum that underlie impulsivity predict compulsive cocaine seeking. Rats that will later compulsively seek cocaine (HC rats) showed, before any drug exposure, lower GM density bilaterally in the ILc **(A)**, revealed by a negative correlation between compulsive cocaine seeking and the ILc density (ILc, [left (•)] rho = −0.386, voxels = 13, cluster-level *p* = .013, [right (Χ)] rho = −0.356, voxels = 130, cluster-level *p* = .014) accompanied by a clear asymmetrical distribution of HC and LC rats in the lower and upper terciles of the population ranked on the ILc density (4 of 7 HC rats were in the lower tercile as opposed to 3 LC rats that instead were predominantly represented in the upper tercile [10 of 16], which only contained 1 HC, χ^2^_4.92_: *p* = .0265) and a difference between groups in the left ventral striatum **(B)** (HC vs. LC rats, voxels = 135, *p* = .003). **(C)** Conversely, HC rats showed higher GM density in the left caudal AI (HC vs. LC rats: voxels = 76, *p* = .019). This structural signature overlapped with that of HI rats which, compared with LI rats, showed lower GM density bilaterally in the ILc **(D)**, the right ventral striatum **(E)**, and the left rostral AI **(F)** (HI vs. LI rats: voxels = 176, *p* = .005; voxels = 397, *p* < .0001 and voxels = 154, *p* = .008, respectively). Scatter plot and bar chart represent mean adjusted GM density from the significant cluster of interest. AI, anterior insula; ALP, active lever press; GM, gray matter; HC, high-compulsivity; HI, high-impulsivity; ILc, infralimbic cortex; L, left hemisphere; LC, low-compulsivity; LI, low-impulsivity; R, right hemisphere.

Next, we sought to define the neural basis of the complex endophenotype predicting compulsive cocaine seeking and the extent to which it mapped onto recent structural and functional neuroimaging studies of the propensity to stimulant use disorder in humans (10,13,53). Voxel-based morphometry analysis of scans carried out after behavioral screening and before any cocaine self-administration (∼postnatal day 228) (Figure 3; Figures S1 and S3) revealed that compared with LC rats, rats destined to compulsively seek cocaine (HC rats) had lower gray matter density in the infralimbic cortex (ILc), as shown by a negative correlation between compulsive cocaine seeking and the ILc density accompanied by an asymmetrical distribution of HC and LC rats in the lower and upper terciles of the population ranked on ILc density (Figure 3A). Rats that were destined to become compulsive also showed lower gray matter density in the left ventral striatum (Figure 3B) but greater gray matter density in the left caudal anterior insula (AI) (Figure 3C). This structural signature was specific to the tendency to seek cocaine compulsively because no such structural differences were observed in relation to baseline cue-controlled cocaine seeking (Figure S10) and overlapped with the neural signature of impulsivity. Therefore, compared with LI rats, HI rats showed lower gray matter density in the ILc (Figure 3D), right ventral striatum (Figure 3E), and left rostral AI (Figure 3F), a neural profile that is consistent with earlier findings (38,39). Together, these data suggest a rostro-caudal functional gradient in the AI mapping onto two different, yet interacting, behavioral manifestations of impulse control deficit, namely impulsivity and compulsivity, respectively (19,39,54, 55, 56, 57).

**Figure 4:**
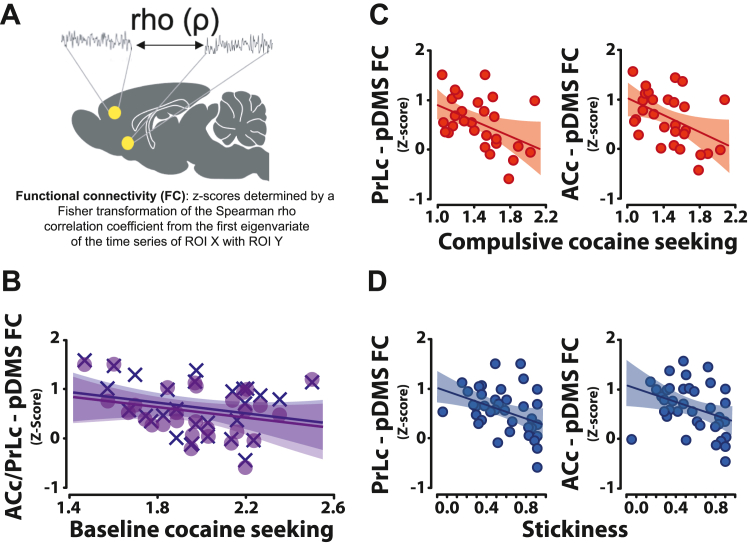
The functional hypoconnectivity of frontostriatal regions that underlies stickiness predicts the vulnerability to develop compulsive cocaine seeking. **(A)** Several prefrontal-striatal regions of interest were assessed for their FC using Spearman’s rho correlation coefficient, displayed here as *z* scores determined by a Fisher transformation of the Spearman rho correlation coefficient from the first eigenvariate of the time series of ROI X with ROI Y. The FC between the ACc (Χ) and PrLc (•) to the pDMS, which was otherwise not significantly associated with cocaine seeking at baseline **(B)**, was in drug-naïve rats inversely proportional to the level of subsequent compulsive cocaine seeking (rho = −0.441, FDR-corrected *p* = .038 and rho = −0.457, FDR-corrected *p* = .030, respectively) **(C)**. The hypofunctionality of these two systems in relation to compulsivity was similar to that observed in relation to stickiness (κ) prior to any drug exposure (rho = −0.463, FDR-corrected *p* = .027 and rho = −0.425, FDR-corrected *p* = .048, respectively) **(D)** (73). ACc, anterior cingulate cortex; FC, functional connectivity; FDR, false discovery rate; pDMS, posterior dorsomedial striatum; PrLc, prelimbic cortex; ROI, region of interest.

The functional coupling signature (Figure 4A; Figures S11C, D and S12) of compulsivity revealed a specific association with corticostriatal networks involved in goal-directed instrumental responding (58,59). The coherence of the coupling strength of the blood oxygen level–dependent response between either the prelimbic cortex (PrLc) or the anterior cingulate cortex (ACc) and the posterior dorsomedial striatum (pDMS) was lower in HC than in noncompulsive rats (Figure 4C) but was not related to baseline cue-controlled cocaine seeking (Figure 4B). Importantly, this hypofunctionality of the PrLc→pDMS, and ACc→pDMS that characterizes compulsive cocaine seeking was also shown to underlie stickiness (Figure 4D), but not impulsivity (Figure S11A, B), possibly reflecting impaired goal-directed behavioral circuitry.

## Discussion

Our findings, based on a prospective longitudinal study of a cohort of outbred male rats, demonstrate that the co-occurrence of high impulsivity and high stickiness traits increases the vulnerability to develop compulsive cocaine seeking. These traits are related to structural and functional connectivity changes in dual neural systems that predict, prior to any drug exposure, the future transition to compulsive cocaine seeking. At the neural systems level, impulsivity was accompanied by structural changes in the ILc, AI, and ventral striatum, consistent with the recognized role of these regions in impulse control (38,39,60); the associated loss of control over cocaine intake (49,61) or inflexible behaviors (62) and stickiness, as indexed by κ, was associated with reduced connectivity strength between dorsomedial areas of the prefrontal cortex (ACc/PrLc) and pDMS. These findings are consistent with previous work showing a negative relationship between the thickness of AI and impulsivity in rats (39) and increased stickiness in human stimulant users (52).

In humans, many studies have revealed structural and functional alterations of similar corticostriatal networks in substance use disorder (10,41,63,64), which may be preexisting vulnerability factors, or may also be the result of chronic drug exposure, or both, making it impossible to disambiguate causality. In contrast, our prospective longitudinal study has enabled resolution of this issue. We showed that the vulnerability to develop compulsive cocaine seeking was predicted by abnormalities in two distinct neural circuitries. One involved decreased functional connectivity between the ACc/PrLc and pDMS and was associated with increased behavioral stickiness. The second involved structural abnormalities in the ILc and ventral striatum, 2 key nodes of an impulsivity network associated with loss of top-down cognitive control (65).

Reduced connectivity between the medial prefrontal cortex and pDMS, a region that has been implicated in goal-directed behavior, including drug seeking (66), is consistent with recent findings of a neurobehavioral endophenotype in this system in humans with stimulant use disorder and their unaffected relatives (53). It invites the hypothesis that preexisting impairments in the goal-directed system lead to inflexible (sticky), habit-prone behavior, which is expressed in compulsive drug seeking (29). This tendency toward behavioral repetition independent of outcome may reflect the default operation of a value-free habit system that complements value-based reinforcement learning (51) and may underlie the profound perseverative tendencies of individuals who are addicted to cocaine, exacerbated by further failures of cognitive control (8,67,68). Thus, preexisting deficits in different circuits contributing to impulse control and flexible, goal-directed behavior may be joint precursors for the emergence of compulsive cocaine seeking.

The transition to severe cocaine use disorder (or dependence) has been reported to occur in only 15% to 20% of cocaine users (47), which is similar to the proportion of outbred rats that developed compulsive cocaine seeking in the current study using an objective cluster analysis approach. This is consistent with previous data showing that only 15% to 20% of male Sprague Dawley or Lister Hooded rats develop compulsive cocaine taking (4,19). Furthermore, the combined statistical power of dimensional analyses and general linear models used here on a cohort of 39 individuals, together with between-subject comparisons of behaviorally characterized groups systematically yielding large to very large effect sizes, confirmed the previously established relationship between impulsivity and compulsivity (19). This approach also allowed us to identify a novel interaction between impulsivity and stickiness in determining vulnerability to compulsive cocaine seeking.

However, a limitation of the current study is that it was confined to male rats to incorporate the results into a large existing dataset on addiction vulnerability that is also mostly confined to male rats (26,69, 70, 71, 72). Although severe cocaine use disorder is more prevalent in men than in women, a necessary next step will be to undertake a longitudinal study with a large cohort of female rats to test the generalizability of these findings.

This identification of a complex neurobehavioral endophenotype for stimulant use disorder has implications for how we approach related psychiatric disorders, including addictions, as a necessary prelude to determining how the development of these fundamental behavioral systems is subject to polygenic and early experiential influences. This knowledge may ultimately help us appreciate how such putative genetic and environmental factors lead to the evidently profound individual differences in susceptibility to compulsive drug-seeking behavior that remain central to understanding and preventing substance use disorders.

The multimodal approach that we have adopted (with its neuroimaging and computational as well as behavioral components) may also represent a more general strategy for modeling the etiology of other neuropsychiatric disorders.

## Supplementary Material

Key Resources Table

## References

[1] American Psychological Association (2013).

[2] Koob G.F., Le Moal M. (2001). Drug addiction, dysregulation of reward, and allostasis. Neuropsychopharmacology.

[3] Koob G.F., Volkow N.D. (2010). Neurocircuitry of addiction. Neuropsychopharmacology.

[4] Fouyssac M., Puaud M., Ducret E., Marti-Prats L., Vanhille N., Ansquer S. (2021). Environment-dependent behavioral traits and experiential factors shape addiction vulnerability. Eur J Neurosci.

[5] Comeau N., Stewart S.H., Loba P. (2001). The relations of trait anxiety, anxiety sensitivity, and sensation seeking to adolescents’ motivations for alcohol, cigarette, and marijuana use. Addict Behav.

[6] Whelan R. (2015). Neuroimaging, addiction and big data: Opportunities and challenges. Neuropsychopharmacology.

[7] Wingo T., Nesil T., Choi J.S., Li M.D. (2016). Novelty seeking and drug addiction in humans and animals: From behavior to molecules. J Neuroimmune Pharmacol.

[8] Jentsch J.D., Taylor J.R. (1999). Impulsivity resulting from frontostriatal dysfunction in drug abuse: Implications for the control of behavior by reward-related stimuli. Psychopharmacol (Berl).

[9] Verdejo-García A., Lawrence A.J., Clark L. (2008). Impulsivity as a vulnerability marker for substance-use disorders: Review of findings from high-risk research, problem gamblers and genetic association studies. Neurosci Biobehav Rev.

[10] Zilverstand A., Huang A.S., Alia-Klein N., Goldstein R.Z. (2018). Neuroimaging impaired response inhibition and salience attribution in human drug addiction: A systematic review. Neuron.

[11] Goldstein R.Z., Volkow N.D. (2011). Dysfunction of the prefrontal cortex in addiction: Neuroimaging findings and clinical implications. Nat Rev Neurosci.

[12] Ersche K.D., Williams G.B., Robbins T.W., Bullmore E.T. (2013). Meta-analysis of structural brain abnormalities associated with stimulant drug dependence and neuroimaging of addiction vulnerability and resilience. Curr Opin Neurobiol.

[13] Ersche K.D., Jones P.S., Williams G.B., Turton A.J., Robbins T.W., Bullmore E.T. (2012). Abnormal brain structure implicated in stimulant drug addiction. Science.

[14] Ersche K.D., Turton A.J., Pradhan S., Bullmore E.T., Robbins T.W. (2010). Drug addiction endophenotypes: Impulsive versus sensation-seeking personality traits. Biol Psychiatry.

[15] Dalley J.W., Theobald D.E., Berry D., Milstein J.A., Lääne K., Everitt B.J., Robbins T.W. (2005). Cognitive sequelae of intravenous amphetamine self-administration in rats: Evidence for selective effects on attentional performance. Neuropsychopharmacology.

[16] Zhukovsky P., Puaud M., Jupp B., Sala-Bayo J., Alsiö J., Xia J. (2019). Withdrawal from escalated cocaine self-administration impairs reversal learning by disrupting the effects of negative feedback on reward exploitation: A behavioral and computational analysis. Neuropsychopharmacology.

[17] Cocker P.J., Rotge J.Y., Daniel M.L., Belin-Rauscent A., Belin D. (2020). Impaired decision making following escalation of cocaine self-administration predicts vulnerability to relapse in rats. Addict Biol.

[18] Belin D., Berson N., Balado E., Piazza P.V., Deroche-Gamonet V. (2011). High-novelty-preference rats are predisposed to compulsive cocaine self-administration. Neuropsychopharmacology.

[19] Belin D., Mar A.C., Dalley J.W., Robbins T.W., Everitt B.J. (2008). High impulsivity predicts the switch to compulsive cocaine-taking. Science.

[20] Tomie A., Brooks W., Zito B., Klein S.B., Mowrer R.R. (1989). Contemporary Learning Theories: Pavlovian Conditioning and the Status of Traditional Learning Theory.

[21] Pohorala V., Enkel T., Bartsch D., Spanagel R., Bernardi R.E. (2021). Sign- and goal-tracking score does not correlate with addiction-like behavior following prolonged cocaine self-administration. Psychopharmacology (Berl).

[22] Everitt B.J., Robbins T.W. (2000). Second-order schedules of drug reinforcement in rats and monkeys: Measurement of reinforcing efficacy and drug-seeking behaviour. Psychopharmacology (Berl).

[23] Cardinal R.N., Parkinson J.A., Hall J., Everitt B.J. (2002). Emotion and motivation: The role of the amygdala, ventral striatum, and prefrontal cortex. Neurosci Biobehav Rev.

[24] Hu Y., Salmeron B.J., Krasnova I.N., Gu H., Lu H., Bonci A. (2019). Compulsive drug use is associated with imbalance of orbitofrontal- and prelimbic-striatal circuits in punishment-resistant individuals. Proc Natl Acad Sci U S A.

[25] Cannella N., Cosa-Linan A., Takahashi T., Weber-Fahr W., Spanagel R. (2020). Cocaine addicted rats show reduced neural activity as revealed by manganese-enhanced MRI. Sci Rep.

[26] Kasanetz F., Deroche-Gamonet V., Berson N., Balado E., Lafourcade M., Manzoni O., Piazza P.V. (2010). Transition to addiction is associated with a persistent impairment in synaptic plasticity. Science.

[27] Belin-Rauscent A., Fouyssac M., Bonci A., Belin D. (2016). How preclinical models evolved to resemble the diagnostic criteria of drug addiction. Biol Psychiatry.

[28] Groman S.M., Massi B., Mathias S.R., Lee D., Taylor J.R. (2019). Model-free and model-based influences in addiction-related behaviors. Biol Psychiatry.

[29] Everitt B.J., Robbins T.W. (2016). Drug addiction: Updating actions to habits to compulsions ten years on. Annu Rev Psychol.

[30] Everitt B.J., Giuliano C., Belin D. (2018). Addictive behaviour in experimental animals: Prospects for translation. Philos Trans R Soc Lond B Biol Sci.

[31] Belin D., Belin-Rauscent A., Everitt B.J., Dalley J.W. (2016). In search of predictive endophenotypes in addiction: Insights from preclinical research. Genes Brain Behav.

[32] Piazza P.V., Deminière J.M., Le Moal M., Simon H. (1989). Factors that predict individual vulnerability to amphetamine self-administration. Science.

[33] Robbins T.W. (2002). The 5-choice serial reaction time task: Behavioural pharmacology and functional neurochemistry. Psychopharmacology (Berl).

[34] Marzuki A.A., Tomic I., Ip S.H.Y., Gottwald J., Kanen J.W., Kaser M. (2021). Association of environmental uncertainty with altered decision-making and learning mechanisms in youths with obsessive–compulsive disorder. JAMA Netw Open.

[35] Fouyssac M., Peña-Oliver Y., Puaud M., Lim N.T.Y., Giuliano C., Everitt B.J., Belin D. (2022). Negative urgency exacerbates relapse to cocaine seeking after abstinence. Biol Psychiatry.

[36] Sawiak S.J., Wood N.I., Williams G.B., Morton A.J., Carpenter T.A. (2009). Voxel-based morphometry in the R6/2 transgenic mouse reveals differences between genotypes not seen with manual 2D morphometry. Neurobiol Dis.

[37] Ashburner J. (2007). A fast diffeomorphic image registration algorithm. Neuroimage.

[38] Caprioli D., Sawiak S.J., Merlo E., Theobald D.E., Spoelder M., Jupp B. (2014). Gamma aminobutyric acidergic and neuronal structural markers in the nucleus accumbens core underlie trait-like impulsive behavior. Biol Psychiatry.

[39] Belin-Rauscent A., Daniel M.L., Puaud M., Jupp B., Sawiak S., Howett D. (2016). From impulses to maladaptive actions: The insula is a neurobiological gate for the development of compulsive behavior. Mol Psychiatry.

[40] Nakama H., Chang L., Fein G., Shimotsu R., Jiang C.S., Ernst T. (2011). Methamphetamine users show greater than normal age-related cortical gray matter loss. Addiction.

[41] Meade C.S., Bell R.P., Towe S.L., Hall S.A. (2020). Cocaine-related alterations in fronto-parietal gray matter volume correlate with trait and behavioral impulsivity. Drug Alcohol Depend.

[42] Cannella N., Cosa-Linan A., Roscher M., Takahashi T.T., Vogler N., Wängler B., Spanagel R. (2017). [18F]-Fluorodeoxyglucose-positron emission tomography in rats with prolonged cocaine self-administration suggests potential brain biomarkers for addictive behavior. Front Psychiatry.

[43] Jia Z., Tang W., Zhao D., Hu G., Li R., Yu S. (2018). Volumetric abnormalities of the brain in a rat model of recurrent headache. Mol Pain.

[44] Grandjean J., Canella C., Anckaerts C., Ayrancı G., Bougacha S., Bienert T. (2020). Common functional networks in the mouse brain revealed by multi-centre resting-state fMRI analysis. Neuroimage.

[45] Benjamini Y., Hochberg Y. (1995). Controlling the false discovery rate: A practical and powerful approach to multiple testing. J R Stat Soc B.

[46] Giuliano C., Belin D., Everitt B.J. (2019). Compulsive alcohol seeking results from a failure to disengage dorsolateral striatal control over behavior. J Neurosci.

[47] Anthony J.C., Warner L.A., Kessler R.C. (1994). Comparative epidemiology of dependence on tobacco, alcohol, controlled substances, and inhalants: Basic findings from the National Comorbidity Survey. Exp Clin Psychopharmacol.

[48] Domi E., Xu L., Toivainen S., Nordeman A., Gobbo F., Venniro M. (2021). A neural substrate of compulsive alcohol use. Sci Adv.

[49] Dalley J.W., Fryer T.D., Brichard L., Robinson E.S., Theobald D.E., Lääne K. (2007). Nucleus accumbens D2/3 receptors predict trait impulsivity and cocaine reinforcement. Science.

[50] Daw N., Delgado M.R., Phelps E.A., Robbins T.W. (2011). Decision Making, Affect, and Learning: Attention and Performance XXIII.

[51] Miller K.J., Shenhav A., Ludvig E.A. (2019). Habits without values. Psychol Rev.

[52] Kanen J.W., Ersche K.D., Fineberg N.A., Robbins T.W., Cardinal R.N. (2019). Computational modelling reveals contrasting effects on reinforcement learning and cognitive flexibility in stimulant use disorder and obsessive-compulsive disorder: Remediating effects of dopaminergic D2/3 receptor agents. Psychopharmacology (Berl).

[53] Ersche K.D., Meng C., Ziauddeen H., Stochl J., Williams G.B., Bullmore E.T., Robbins T.W. (2020). Brain networks underlying vulnerability and resilience to drug addiction. Proc Natl Acad Sci U S A.

[54] Grodin E.N., Cortes C.R., Spagnolo P.A., Momenan R. (2017). Structural deficits in salience network regions are associated with increased impulsivity and compulsivity in alcohol dependence. Drug Alcohol Depend.

[55] Fernández-Serrano M.J., Cesar Peraleslópez J., Moreno-López L., Santos-Ruiz A., Pérez-García M., Verdejogarcía A. (2012). [Impulsivity and compulsivity in cocaine dependent individuals]. Adicciones.

[56] Grant J.E., Potenza M.N. (2006). Compulsive aspects of impulse-control disorders. Psychiatr Clin North Am.

[57] McElroy S.L., Phillips K.A., Keck P.E. (1994). Obsessive compulsive spectrum disorder. J Clin Psychiatry.

[58] Yin H.H., Ostlund S.B., Knowlton B.J., Balleine B.W. (2005). The role of the dorsomedial striatum in instrumental conditioning. Eur J Neurosci.

[59] Balleine B.W., O’Doherty J.P. (2010). Human and rodent homologies in action control: Corticostriatal determinants of goal-directed and habitual action. Neuropsychopharmacology.

[60] Chudasama Y., Passetti F., Rhodes S.E., Lopian D., Desai A., Robbins T.W. (2003). Dissociable aspects of performance on the 5-choice serial reaction time task following lesions of the dorsal anterior cingulate, infralimbic and orbitofrontal cortex in the rat: Differential effects on selectivity, impulsivity and compulsivity. Behav Brain Res.

[61] Rotge J.Y., Cocker P.J., Daniel M.L., Belin-Rauscent A., Everitt B.J., Belin D. (2017). Bidirectional regulation over the development and expression of loss of control over cocaine intake by the anterior insula. Psychopharmacology (Berl).

[62] Barker J.M., Taylor J.R., Chandler L.J. (2014). A unifying model of the role of the infralimbic cortex in extinction and habits. Learn Mem.

[63] Tomasi D., Volkow N.D. (2013). Striatocortical pathway dysfunction in addiction and obesity: Differences and similarities. Crit Rev Biochem Mol Biol.

[64] Vollstädt-Klein S., Wichert S., Rabinstein J., Bühler M., Klein O., Ende G. (2010). Initial, habitual and compulsive alcohol use is characterized by a shift of cue processing from ventral to dorsal striatum. Addiction.

[65] Dalley J.W., Robbins T.W. (2017). Fractionating impulsivity: Neuropsychiatric implications. Nat Rev Neurosci.

[66] Murray J.E., Belin D., Everitt B.J. (2012). Double dissociation of the dorsomedial and dorsolateral striatal control over the acquisition and performance of cocaine seeking. Neuropsychopharmacology.

[67] Ersche K.D., Barnes A., Jones P.S., Morein-Zamir S., Robbins T.W., Bullmore E.T. (2011). Abnormal structure of frontostriatal brain systems is associated with aspects of impulsivity and compulsivity in cocaine dependence. Brain.

[68] Ersche K.D., Roiser J.P., Robbins T.W., Sahakian B.J. (2008). Chronic cocaine but not chronic amphetamine use is associated with perseverative responding in humans. Psychopharmacology (Berl).

[69] Augier E., Barbier E., Dulman R.S., Licheri V., Augier G., Domi E. (2018). A molecular mechanism for choosing alcohol over an alternative reward. Science.

[70] Cannella N., Halbout B., Uhrig S., Evrard L., Corsi M., Corti C. (2013). The mGluR2/3 agonist LY379268 induced anti-reinstatement effects in rats exhibiting addiction-like behavior. Neuropsychopharmacology.

[71] Singer B.F., Fadanelli M., Kawa A.B., Robinson T.E. (2018). Are cocaine-seeking “habits” necessary for the development of addiction-like behavior in rats?. J Neurosci.

[72] Chen B.T., Yau H.J., Hatch C., Kusumoto-Yoshida I., Cho S.L., Hopf F.W., Bonci A. (2013). Rescuing cocaine-induced prefrontal cortex hypoactivity prevents compulsive cocaine seeking. Nature.

[73] Paxinos G., Watson C. (2013).

